# PRIMA-1 targets the vulnerability of multiple myeloma of deregulated protein homeostasis through the perturbation of ER stress via p73 demethylation

**DOI:** 10.18632/oncotarget.11241

**Published:** 2016-08-12

**Authors:** Phaik Ju Teoh, Chonglei Bi, Chirackal Sintosebastian, Liang Seah Tay, Rafael Fonseca, Wee Joo Chng

**Affiliations:** ^1^ Cancer Science Institute Singapore, National University of Singapore, Singapore; ^2^ Department of Hematology-Oncology, Mayo Clinic, Scottsdale, Arizona, USA; ^3^ Department of Medicine, Yong Loo Lin School of Medicine, National University of Singapore, Singapore; ^4^ Department of Haematology-Oncology, National University Cancer Institute, Singapore

**Keywords:** multiple myeloma, PRIMA-1, ER stress, p73, bortezomib

## Abstract

Despite therapeutic advancement, multiple myeloma (MM) remains incurable with drug resistance being one of the main challenges in the clinic. Myeloma cells possess high protein secretory load, leading to increased intracellular endoplasmic reticulum (ER) stress. Hence, they are vulnerable to further perturbation to its protein homeostasis. In studying the therapeutic mechanism of PRIMA-1 (mutant-p53-reactivating-agent), we uncovered its novel p53-independent-mechanism that can be exploited for myeloma. Despite its inability in restoring the wild type-p53 protein conformation and transcriptional function in the mutant-p53-human-myeloma-cells, PRIMA-1 was efficacious against myeloma cells with differential p53 genotypes. Strikingly, cells without p53 expression demonstrated highest drug sensitivity. Genome-wide gene-expression analysis revealed the involvement of ER stress/UPR-pathway in inducing PRIMA-1-toxicity. UPR markers, HSP70, CHOP and GADD34, were significantly up-regulated, concomitantly with the induction of apoptosis. Furthermore, there was a global attenuation of protein synthesis, correlated with phospho-eIF2a up-regulation. Mechanistically, we identified that PRIMA-1 could cause the demethylation of *TP73*, through DNMT1 depletion, to subsequently enhance UPR. Of clinical significance, we observed that PRIMA-1 had additive therapeutic effects with another UPR-inducing-agent, bortezomib. Importantly, it can partially re-sensitize bortezomib-resistant cells to bortezomib. Given that MM is already stressed at the baseline in the ER, our results implicated that PRIMA-1 is a potential therapeutic option in MM by targeting its Achilles heel.

## INTRODUCTION

Multiple myeloma (MM) is the second most common haematological malignancy characterized by abnormal proliferation of antibody-producing-plasma cells [1]. Although the emergence of novel therapeutics such as bortezomib has revolutionized the treatment scene in MM, drug resistance remains inevitable and only about 10% of patients have a 10-year survival rate [2, 3]. In particular, patients harbouring *TP53* deletion/mutation have very poor prognosis [4–6]. Importantly, we have previously shown that in MM cases with hemizygous 17p13(del), the p53 pathway is already attenuated [7], rendering these patients to be highly resistant to standard therapeutics [6, 8]. Restoration of the functional p53 signalling is therefore an important therapeutic strategy in these high-risk disease.

PRIMA-1 (p53 reactivation and induction of massive apoptosis) is a small molecular drug that was functionally discovered to reactivate mutant p53 by restoring its WT (wild type) protein conformation, transcriptional activity and its ultimate tumour suppressive properties [9, 10]. Its classical function in reactivating mutant p53 is well established in human cancers, including breast, lung, thyroid and ovarian cancer [11–14]. PRIMA-1 was able to reconstitute p53 activity by inducing the transcription of various downstream targets such as p21, BAX, PUMA and NOXA and a consequent mutant-p53-dependent apoptosis [11, 12, 15].

Due to the promising results generated from previous *in vivo* and *in vitro* studies, PRIMA-1Met (the more potent derivative) has made its way to Phase I/II clinical trials [15]. The drug was generally well tolerated with the cancer cells harvested from several patients were noted to undergo cell cycle arrest, increased apoptosis, and up-regulation of p53 target genes.

With this in mind, we tested the therapeutic effects of PRIMA-1 in a panel of MM cell lines of various p53 functional status. Consistent with some recent reports [16, 17], we found that PRIMA-1 can effectively kill myeloma cells independently of their p53 genotype. Intriguingly, its highest potency was exhibited in cells with zero p53 expression. High-throughput microarray analysis and subsequent investigations then unveiled an important novel mechanism by which PRIMA-1 could kill MM cells, which is via the activation of endoplasmic reticulum (ER) stress or Unfolded Protein Response (UPR) pathway that was mediated by p73 demethylation. Given that myeloma cells are by nature vulnerable to additional ER stress due to its extensive protein burden *in vivo*, targeting the ER stress pathway may be an attractive way to combat the disease. The novel mechanism of action uncovered in our study suggests that PRIMA-1 and its derivative may be used in myeloma to exploit this vulnerability.

## RESULTS

### PRIMA-1's non-canonical role in MM

**Figure 1:**
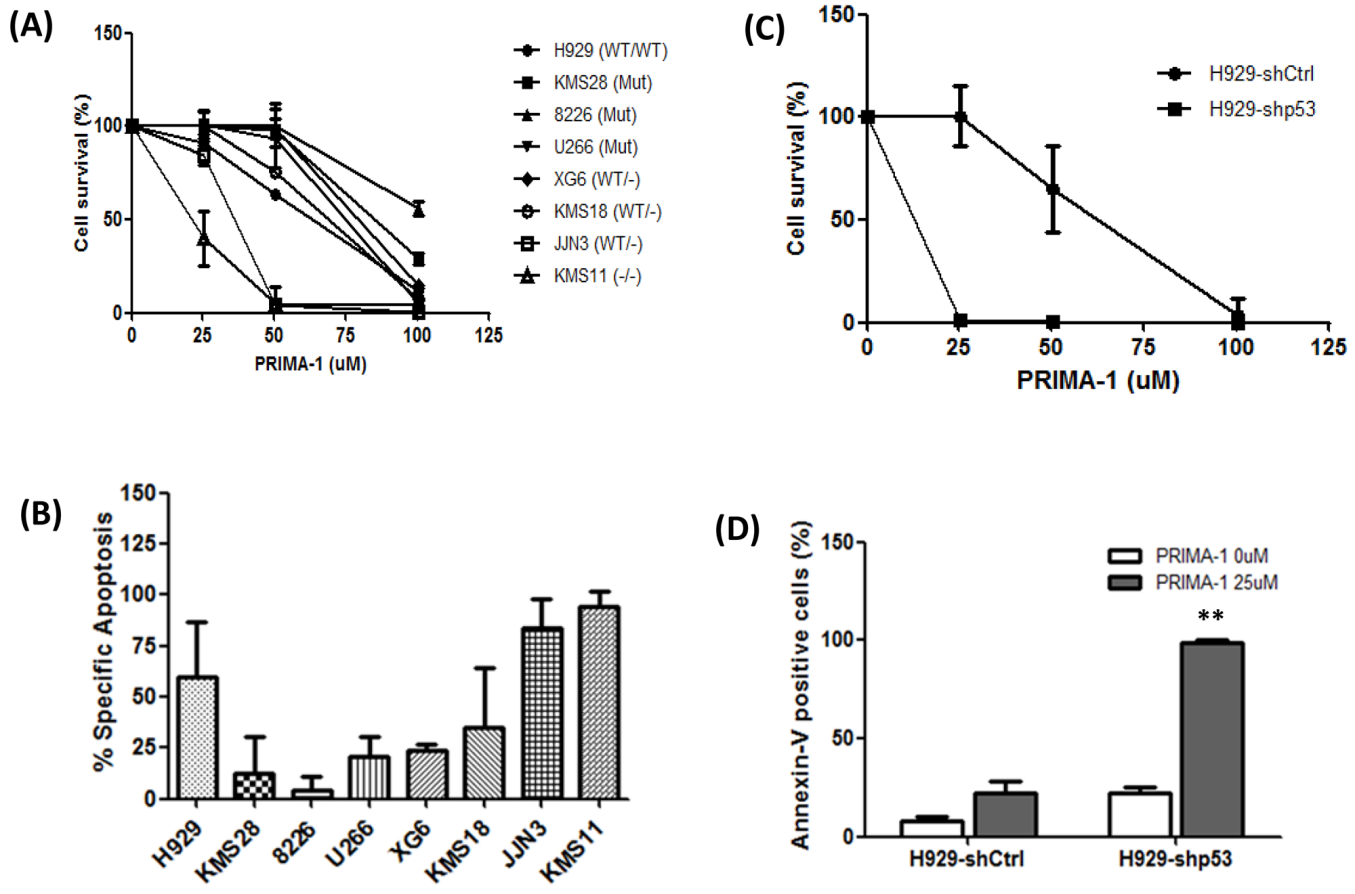
PRIMA-1's non-canonical role in MM **A.** MTS assay showing cell viability of all the PRIMA-1-treated HMCLs relative to DMSO control (48 hours). **B.** Cells were treated with DMSO or 50uM PRIMA-1 for 48 hours and were subjected to flow cytometry analysis for the detection of annexin-V-positive cells. Data was presented as percentage specific apoptosis. Percentage specific apoptosis was calculated using the equation: % specific apoptosis¼ (Test- control)*100/100-control. **C.** MTS assay showing cell viability of H929-p53 knockdown isogenic cell lines upon PRIMA-1 treatment for 48 hours. **D.** Cells were treated with DMSO or PRIMA-1 for 48 hours and percentage annexin-V-positive cells were quantified with flow cytometer. ** p<0.01

Consistent with previous reports in MM [16, 17], we found that PRIMA-1 was able to induce growth inhibition of HMCLs, irrespective of their p53 functional status (Figure 1A; IC50s in Supplementary Table S1). Interestingly, JJN3 and KMS11, both with zero p53 mRNA and protein (Supplementary Figure S1), were noted to be significantly more sensitive to PRIMA-1 than other HMCLs, including those with p53 mutation. Annexin-V assay also showed the highest degree of apoptosis in these two HMCLs (Figure 1B). Treatment with 50uM of PRIMA-1 only managed to kill less than 60% of the cells in the remaining HMCLs. Cell cycle analysis also revealed the highest sub-G1 population in PRIMA-1-treated KMS11 and JJN3 (Supplementary Figure S2). When we treated the same set of HMCLs with its derivative, PRIMA-1Met, all of them experienced growth inhibition with generally lower IC50s than its parental drug (Supplementary Table S2). Consistently, the IC50s in JJN3 and KMS11 were also found to be much lower than other HMCLs. Collectively, these data strongly suggest that p53 (be it WT or mutant) is dispensable for PRIMA-1 treatment and that its cytotoxicity was most prevalent in the absence of p53.

To further validate this finding, isogenic p53-knockdown-NCI-H929 (sh-Ctrl and sh-p53) [7] were similarly treated. Indeed, the silencing of p53 rendered the cells more sensitive towards PRIMA-1 as reflected by a dramatic reduction in cell survival (Figure 1C) and significant increase in the percentage of apoptosis (Figure 1D), thus, further emphasizing the better efficiency of PRIMA-1 in a p53-null background.

In view of the high IC50 in the p53-mutant HMCLs, we also examined if PRIMA-1 has any direct effects on the protein structure of the mutant-p53 proteins. By using a p53-WT-conformation-specific antibody (PAb 1620), we identified that PRIMA-1 was not able to restore the p53-WT structure (Supplementary Figure S3A). Concordantly, no changes were seen in the expression levels of p53 transcriptional targets, namely, p21, MDM2 and PUMA, even when they were treated up to 100uM, indicating that the transcriptional function of the mutant proteins was not reinstated (Supplementary Figure S3B).

In other words, the canonical role of PRIMA-1 in rescuing the WT function of mutant-p53 was not applicable in MM. Since it is found that the p53-null cells were most sensitive to the drug, this only implied that factors other than p53 was prevailing in PRIMA-1's mechanism of action in MM.

### PRIMA-1 treatment induces the activation of ER stress pathway/UPR

**Figure 2:**
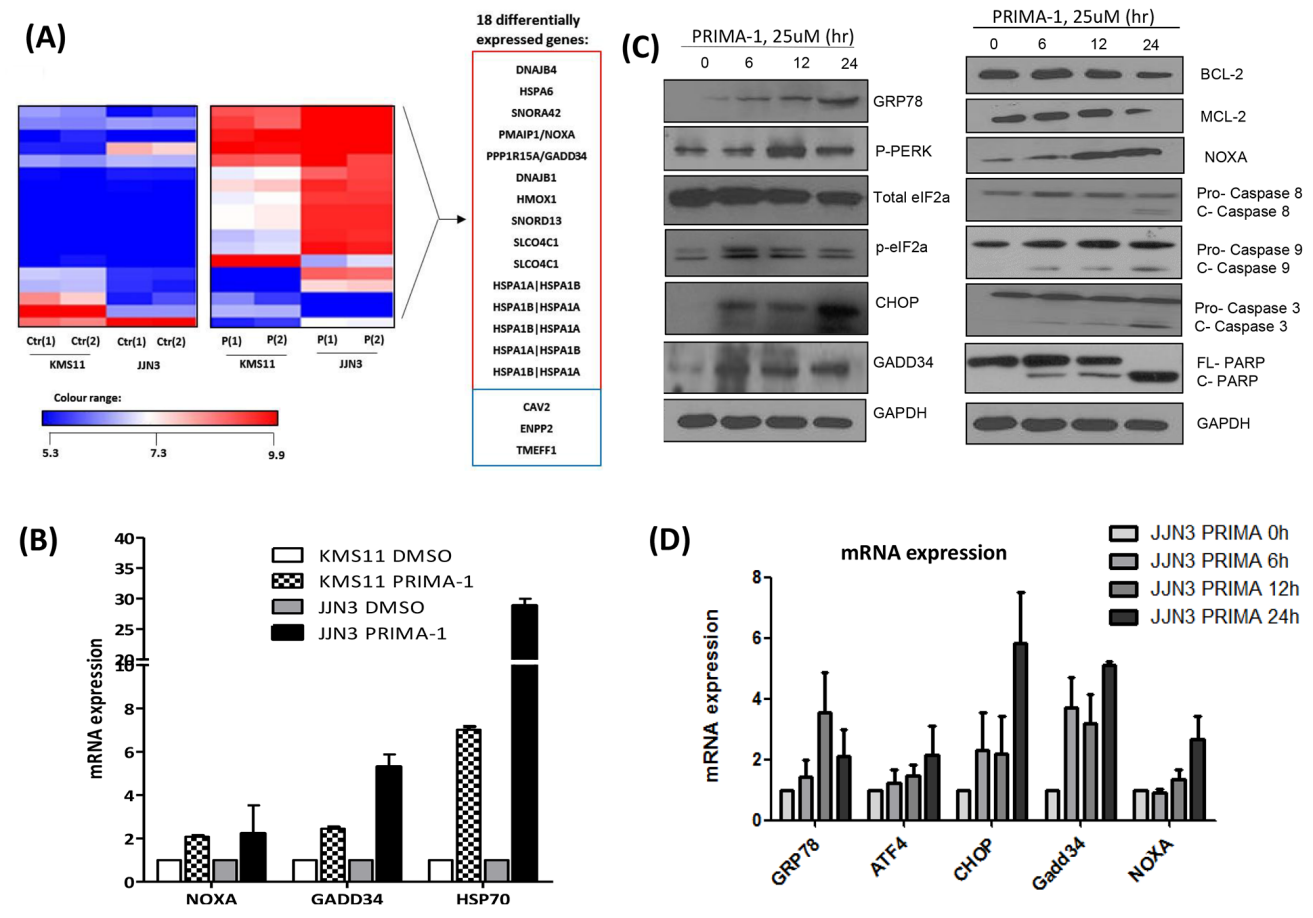

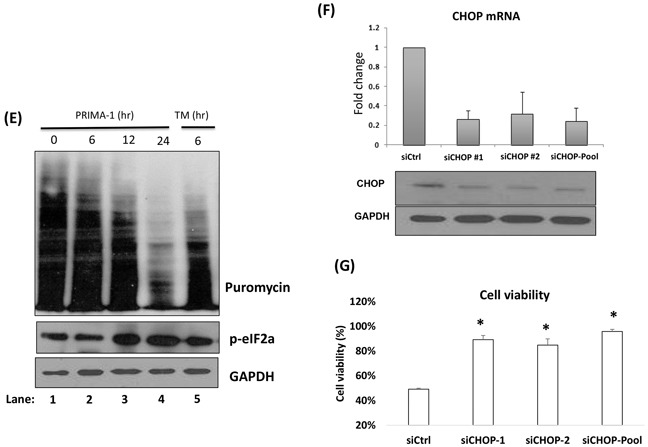
PRIMA-1 treatment induces the activation of ER stress pathway/UPR **A.** Heat map demonstrating the differentially expressed genes of the PRIMA-1 versus DMSO-treated samples in JJN3 and KMS11 (Fold change = 2, p < 0.05). The experiment was conducted in duplicates. Ctr(1): DMSO-treated sample 1, Ctr(2): DMSO-treated sample 2, P(1): PRIMA-1-treated sample 1, P(2): PRIMA-1-treated sample 2. Genes in red box: upregulated, genes in blue box: downregulated. **B.** Real time-PCR validation of GEP analysis revealed upregulation of NOXA, GADD34 and HSP70 upon 25uM PRIMA-1 treatment (24 hours). **C.** Western blot analysis of various ER stress and apoptotic markers in JJN3 treated with PRIMA-1 (25uM) at different time points. **D.** Real time-PCR analysis of ER stress markers in JJN3 treated with PRIMA-1 25uM at different time points. **E.** Newly synthesized proteins were labelled with puromycin in JJN3 cells after drug treatment (25uM) at different time points. TM: Tunicamycin. **F.** JJN3 was transfected with 100nM siCtrl or two independent sequences of siCHOP or combined sequences of siCHOP #1 and siCHOP #2 for 24 hours. mRNA and protein were isolated to check for knockdown efficiency. **G.** 24 hours post-CHOP knockdown, JJN3 cells were treated with 25uM PRIMA-1 for another 48 hours. Cell viability was quantified by MTS assay. * : p<0.05.

To elucidate the p53-independent mechanism of PRIMA-1, we performed a systematic genome-wide GEP analysis in JJN3 and KMS11. Comparing DMSO versus PRIMA-1-treated samples, we noted a series of differentially expressed genes encoding the stress and heat-shock proteins alongside regulators of cell proliferation, protein folding and lipid metabolism (Fold change ≥2, p <0.05). Eighteen differentially expressed genes were common in both HMCLs (Figure 2A and Supplementary Figure S4). When validated by PCR, important cancer-related genes such as NOXA (apoptotic gene), GADD34 (cell cycle regulator) and HSPA1A, HSPA1B (encoding HSP70 proteins) were up-regulated by at least two folds upon PRIMA-1 treatment (Figure 2B).

Gene ontology analysis revealed two interesting signalling pathways that were significantly enriched upon drug treatment, namely, the unfolded and misfolded protein responses (Supplementary Table S3). Since NOXA, GADD34 and HSP70 have also been closely associated with unfolded protein accumulation [18, 19], these data strongly support the involvement of UPR in mediating PRIMA-1 toxicity in MM.

To validate our hypothesis, various ER stress markers were assessed using Western blot analysis. Indeed, GRP78 (HSP70 family member), necessary for binding to aggregated unfolded/misfolded proteins in ER, showed increased expression upon treatment by PRIMA-1 in a time-dependent manner. In addition, PERK and eIF2a were phosphorylated, and the downstream elements, CHOP and GADD34, were consequently upregulated, all of which are hallmarks of ER stress induction (Figure 2C). This led to the modulation of the pro-apoptotic (NOXA) and anti-apoptotic (BCL-2 and MCL-1) protein expression, ultimately triggering a cascade of apoptosis involving both intrinsic (caspase-9 cleavage) and extrinsic (caspase-8 cleavage) signalling. Accordingly, PRIMA-1 treatment also caused the induction of UPR markers at the transcript level (Figure 2D).

As p-eIF2a functions to momentarily attenuate protein translation for the repair of ER congestion to commence [20], we examined if PRIMA-1 was able to stall protein production by performing the SUnSET assay, a dynamic non-radioactive method utilizing puromycin to label neo-proteins [21]. PRIMA-1 treatment resulted in a gradual reduction of global protein synthesis, with a concomitant increase of p-eIF2a expression (Figure 2E). This observation was replicated in tunicamycin-treated-fraction (Figure 2E, lane 5) (tunicamycin is a classical ER stress inducer), thus validating the UPR-inducing effects of PRIMA-1.

The hallmark of UPR is that cells which are subjected to insurmountable ER stress would switch from adaptation to apoptotic state. CHOP has been shown to be the specific factor mediating this ER stress-induced apoptosis [22, 23]. In line with this, we proceeded to knockdown CHOP (Figure 2F). Indeed, depletion of CHOP resulted in a significantly lower sensitivity to PRIMA-1, as demonstrated by the higher cell viability in these cells relatively to the siCtrl cells (Figure 2G). This finding further confirms that PRIMA-1 cytotoxicity was in part mediated through the UPR-death inducing pathway.

The other sensitive HMCL, KMS11 also displayed an activated UPR (Supplementary Figure S5A), implicating that observation in JJN3 was not a cell line-specific effect. The less sensitive HMCLs such as NCI-H929 (p53-WT) and U266 (p53-mutant) displayed a mild degree of UPR activation, and required a much higher concentration of PRIMA-1 (~50-100uM) to manifest this phenotype (Supplementary Figure S5B). This further corroborates the importance of UPR activation in mediating PRIMA-1 cytotoxicity in myeloma cells.

### PRIMA-1 activated PERK, suppressed IRE1 and has no significant effect on ATF6 arms of UPR

Because UPR constitutes three inter-linked networks mediated by transmembrane protein sensors, namely, IRE1, PERK and ATF6 [20], we sought to know which one is the main target of PRIMA-1. Our earlier results suggested that the PERK network was involved, as shown by the induction of its downstream elements, such as p-PERK, p-eIF2a, ATF4, CHOP and GADD34. CHOP (transcription target of ATF4) knockdown also diminished PRIMA-1-induced-cytotoxicity.

**Figure 3:**
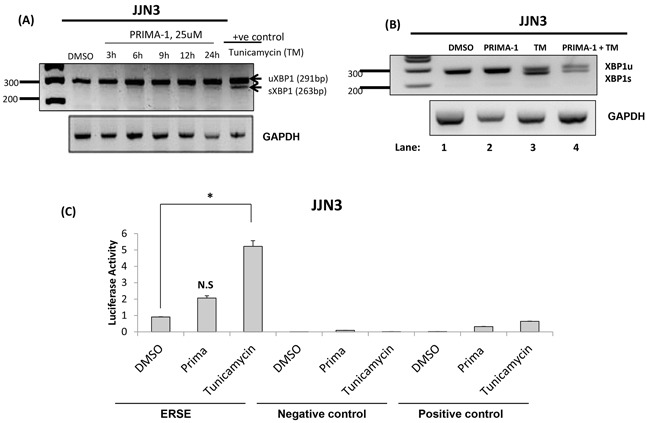
PRIMA-1 activated PERK, suppressed IRE1 and has no significance on ATF6 arms of UPR **A.** JJN3 cells were treated with 25uM PRIMA-1 at the indicated time points. RT-PCR was performed with specific XBP1 primers. 10ug/mL tunicamycin treatment for 6 hours was used as a positive control. uXBP1: unspliced XBP1, sXBP1: spliced XBP1. **B.** JJN3 was treated with either single or combined agents of 25uM PRIMA-1 and 10ug/mL tunicamycin (TM) for 8 hours and RT-PCR was performed. **C.** Luciferase activity displayed by JJN3 when treated with either 25uM PRIMA-1 or 10ug/mL tunicamycin, relatively to DMSO control. N.S: Non-significant; * : p< 0.05. Negative control readings are from cells transfected with an empty vector with firefly luciferase activity. Positive control cells are transfected with the same vectors as the negative control but with an additional monster-GFP vector (for monitoring the transfection efficiency).

XBP1 splicing is the hallmark for IRE1 activation and has been reported to be an early event in UPR [24]. Figure 3A showed that XBP1 was undergoing only a subtle splicing at the 12^th^ and 24^th^ hour, as demonstrated by faint XBP1s bands. In contrast, tunicamycin treatment of a mere 6 hours was sufficient to create an intense splicing. The IRE1/XBP1 route was described to be crucial for differentiation and survival of plasma cells [25, 26], thus, we postulated that PRIMA-1 may be exerting its anti-myeloma role by inhibiting the pro-survival effects of IRE1/XBP1. Indeed, we observed that PRIMA-1 treatment could partially rescue tunicamycin-induced-XBP1s (Figure 3B, lane 4), an indication of the suppression of IRE1 activity.

On the other hand, ATF6 reporter assay revealed a non-significant increase of luciferase activity upon PRIMA-1 treatment (Figure 3C), suggesting that ATF6 network may not be important in PRIMA-1's mechanism of action.

Taken together, these assays denoted that PRIMA-1 may be exerting its cytotoxicity by inhibiting the pro-survival arm of IRE1 to amplify the pro-apoptotic response from PERK activation.

### High basal intracellular ER stress is important for PRIMA-1-induced-toxicity

Because MM plasma cells must be able to sustain high intracellular ER stress for survival and proliferation due to its high secretory load [20, 27], we postulated that cells with a higher baseline ER stress (lower threshold for UPR induction) would be more easily sensitized to PRIMA-1-induced-apoptosis.

**Figure 4:**
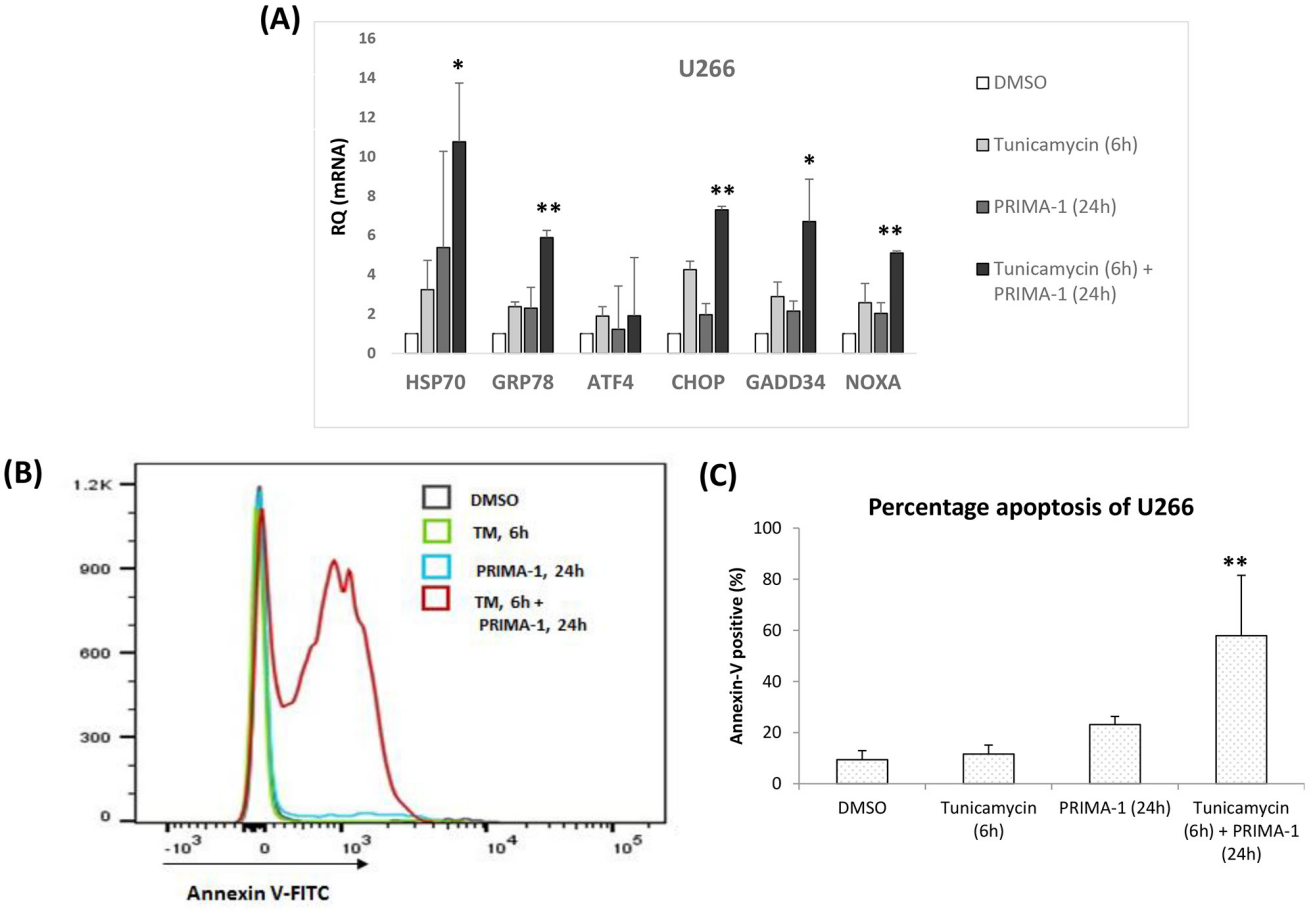
High basal intracellular ER stress was important for PRIMA-1-induced toxicity **A.** mRNA expression of ER stress markers after U266 was treated with either tunicamycin or PRIMA-1 or combination of both. For combination treatment, U266 was pre-treated with 10ug/mL tunicamycin for 6 hours to induce unfolded protein accumulation. Cells were washed twice with PBS to rid of tunicamycin before fresh PRIMA-1 was added to the cells for another 24 hours. **B.** Overlayed-histogram of annexin-V-positivity of U266 cells after indicated treatments as analysed on flow cytometer. Analysis was done on Flowjo (Ashland, Oregon, USA). **C.** Quantification of annexin-V positive cells of U266 after indicated treatments. ** p<0.01

To prove this hypothesis, we utilised a less sensitive HMCL, U266. Firstly, we heightened their basal ER stress level prior to PRIMA-1 treatment. Tunicamycin, is known to disrupt N-linked glycosylation of nascent proteins [28], thus, was used to induce unfolded protein accumulation. As shown in Figure 4A, single treatment of either tunicamycin or PRIMA-1 triggered only a marginal UPR activation, as indicated by increased of two to three folds of UPR markers. This corresponded with the minimal apoptosis response observed in the cells of single treatments (Figure 4B and 4C). On the other hand, pre-treatment of U266 with tunicamycin essentially sensitized the cells to the subsequent PRIMA-1 exposure, whereby a significant increase in apoptosis was recorded (Figure 4B and 4C). This is also in keeping with the expression profile of the UPR markers which showed up-regulation of five to 10 folds when the tunicamycin-pre-treated cells were subsequently incubated with PRIMA-1 (Figure 4A). Therefore, this indicates that inflating the baseline ER stress level by tunicamycin prior to PRIMA-1 treatment could lead to the intensification of UPR, culminating in cellular toxicity. This finding clearly demonstrated the importance of basal ER stress level in bringing about effective apoptosis in response to PRIMA-1 treatment.

### PRIMA-1-induced-p73 led to the enhancement of UPR

Because PRIMA-1 was more toxic in the absence of p53, we hypothesized that p73, being its homologue, may act as a surrogate TSG (tumor suppressor gene). Importantly, Saha et al. has previously highlighted the role of p73 in PRIMA-1-induced-apoptosis [16]. Moreover, p73 has also been associated with elevation of intracellular ER stress through functional incorporation of scotin [29]. Thus, it was highly relevant to interrogate if p73 was associated with PRIMA-1-induced-UPR in myeloma.

**Figure 5:**
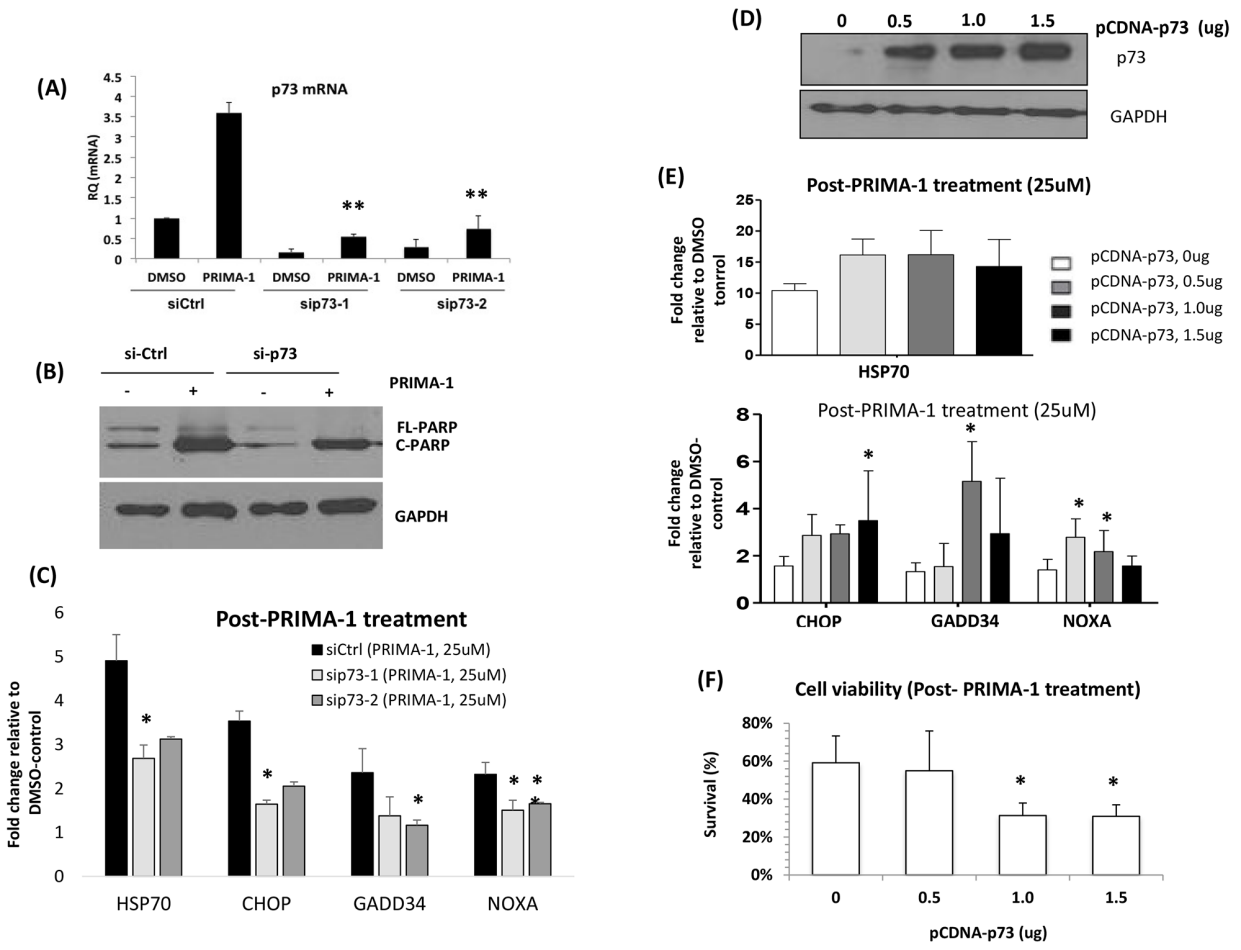
PRIMA-1-induced-p73 led to the enhancement of UPR **A.** JJN3 was transfected with two different siRNAs against *TP73.* At 24 hours post-transfection, the cells were treated with either DMSO control or PRIMA-1 (25uM) for another 24 hours. *TP73* mRNA level was checked via qRT-PCR at 24 hours post-treatment. **B.** JJN3 was transfected with non-targeting siRNA (si-Ctrl) and sip73-1 for 24 hours. Cells were washed and treated with either DMSO or PRIMA-1 for another 24 hours and apoptosis reaction was shown as PARP cleavage (C-PARP). **C.** mRNAfold change level of ER stress markers of siCtrl and sip73-transfected cells after PRIMA-1 treatment, relative to DMSO control. **D.** p73 protein level in JJN3 after transfection with different doses of overexpression plasmid pCDNA-p73 for 48 hours. **E.** JJN3 cells were transfected with the indicated amount of pCDNA-p73 for 48 hours and was subsequently treated with 25uM PRIMA-1 (24 hours). Fold change level of the mRNA expression of various ER stress markers after PRIMA-1 treatment was calculated by normalizing against DMSO control. **F.** Cell viability of JJN3 overexpressed with p73 after 48 hours of 25uM PRIMA-1 treatment. *p<0.05, ** p<0.01

In concordance with the literature, we also found distinct increase of p73 at both the mRNA and protein levels upon PRIMA-1 treatment (Supplementary Figure S6A). In the range of HMCLs tested, p73 is generally low at the basal level, however, we can see a trend whereby cells with higher sensitivity to PRIMA-1 (JJN3 and KMS11) have relatively higher levels of p73 mRNA as compared to cells with lower sensitivity (Supplementary Figure S6B). At the protein level, even though p73 was barely detectable at the baseline in both JJN3 and KMS11, its expression was markedly upregulated upon PRIMA-1 treatment. All other cell lines, albeit seem to also demonstrate an increased expression of p73 in response to PRIMA-1 treatment, the upregulation was either subtle or almost negligible than in the sensitive cell lines (Supplementary Figure S6C), concurring that p73 has an important role in mediating PRIMA-1-induced-toxicity. As expected, loss of p73 expression via siRNA knockdown compromised its induction upon drug treatment (Figure 5A). Lower rate of p73 induction indicates that its expression level was not sufficient to trigger the downstream effects.

Importantly, silencing p73 did not only confer the cells a compromised apoptotic response (lesser c-PARP) (Figure 5B), but the PRIMA-1-induced-upregulation of the ER stress markers were also significantly rescued (Figure 5C). In turn, p73 overexpression (Figure 5D) resulted in an enhancement of the expression level of UPR markers (Figure 5E), accompanied by greater growth inhibition (Figure 5F). These findings implicate the role of p73 in mediating the ER activity and in sustaining the UPR required for the cytotoxicity of PRIMA-1 in MM.

### PRIMA-1-induced-p73 was associated with demethylation of *TP73*

**Figure 6:**
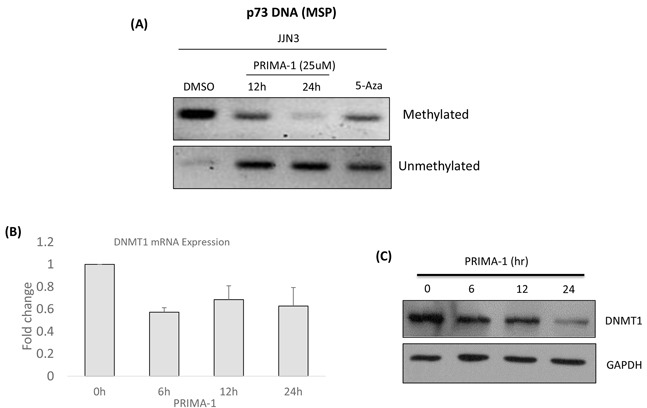
PRIMA-1-induced-p73 was associated with demethylation of *TP73* **A.** Methylation specific PCR was performed after the cells were treated with either DMSO, PRIMA-1 (25uM) or 5-Aza (2uM, 24 hours). Methylated and unmethylated p73 promoter specific primers were used for PCR amplification. 5-Aza: 5-Azacytidine (positive control). **B.** mRNA expression of DNMT1 after PRIMA-1 (25uM) treatment at the indicated time points. **C.** Protein expression of DNMT1 after PRIMA-1 treatment (25uM) at the indicated time points.

Because *TP73* was reported to be commonly silenced by promoter hypermethylation in cancers [30, 31], we proceeded to check if the p73 up-regulation by PRIMA-1 was mediated by demethylation. To clarify this hypothesis, methylation specific PCR (MSP) analysis was performed and indeed, methylated *TP73* showed consistent down-regulation with a simultaneous up-regulation of its demethylated counterpart, upon PRIMA-1 treatment (Figure 6A). Concordantly, there was a reduction of DNA methyltransferase 1 (DNMT1) at both the mRNA (Figure 6B) and protein (Figure 6C) levels, suggesting that there was an association of PRIMA-1-induced-*TP73* demethylation through the attenuation of DNMT1 activity, leading to downstream activation of UPR.

Besides DNMT1, the expression levels of DNMT3A was also checked upon PRIMA-1 treatment. There was a slight downregulation (~20%) of DNMT3A transcript level at the 6^th^ and 12^th^ hour (Supplementary Figure S6D) but the downtrend was abolished come 24^th^ hour. Based on these results, it may indicate that both DNMT1 and DNMT3A may act collaboratively in the early time point but complete demethylation of the target gene was mediated predominantly by DNMT1.

### Clinical significance of PRIMA-1 treatment

**Figure 7:**
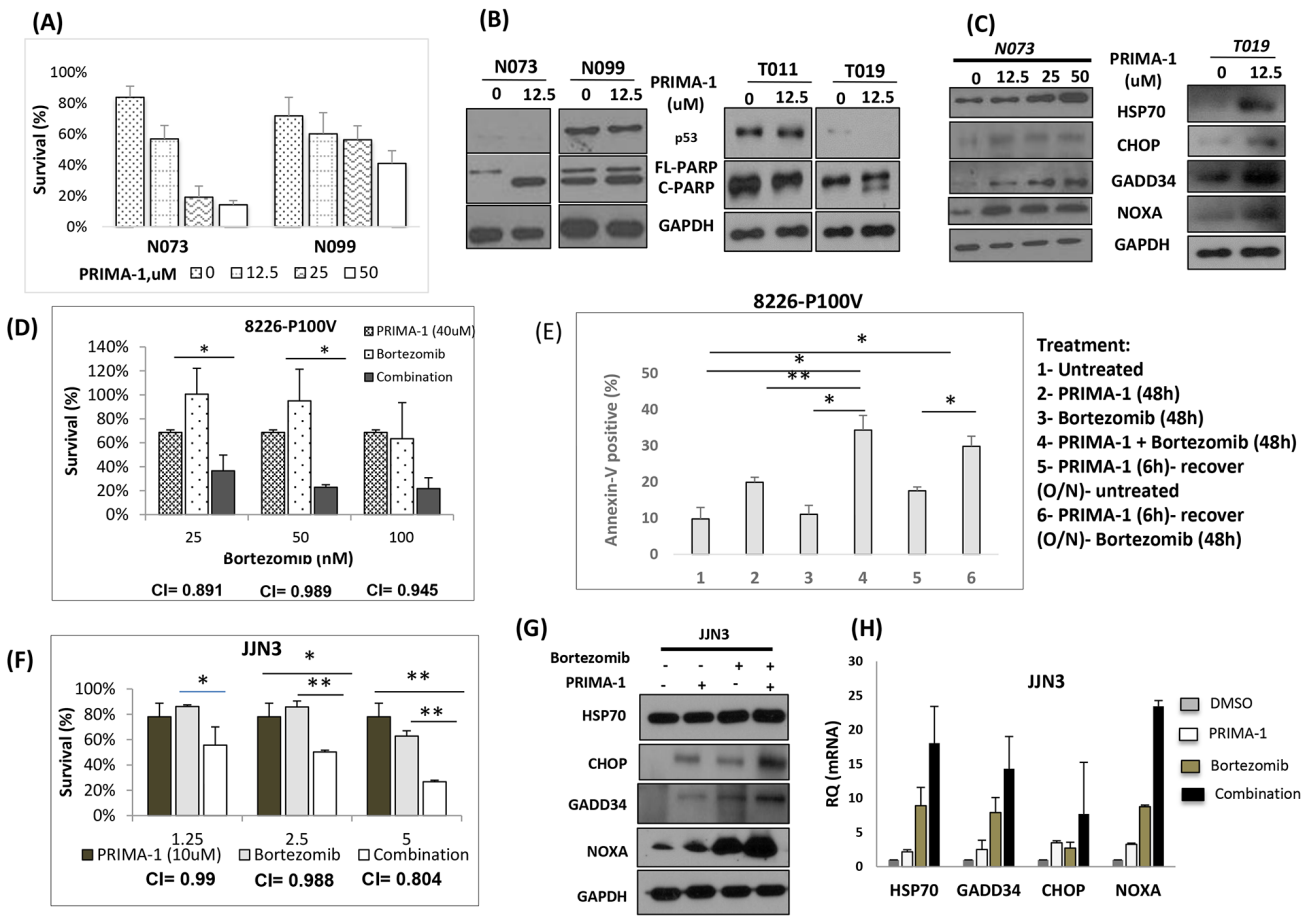
Clinical significance of PRIMA-1 treatment **A.** Cell viability of primary patient samples when they were treated with increasing dosage of PRIMA-1 for 48 hours. **B.** Patient samples were treated with PRIMA-1 and protein expression of p53 and PARP was examined by Western blot analysis. **C.** Samples from patient N073 and T019 were treated with PRIMA-1 for 24 hours and cells were harvested for Western blot analysis probing for HSP70, CHOP, GADD34 and NOXA. **D.** Cell viability of 8226-P100V after treatment with either a fixed dosage of PRIMA-1 (40uM), increasing dosage of bortezomib (25nM, 50nM, 100nM) or combination of PRIMA-1 40uM with the corresponding bortezomib dosage on the x-axis. **E.** Percentage specific apoptosis of P100V after PRIMA-1 or/and bortezomib treatment. 1- Untreated, 2- PRIMA-1 (48h), 3-Bortezomib (48h), 4- Combination of PRIMA-1 and Bortezomib (48h), 5- To test for re-sensitization to bortezomib, P100V were treated with PRIMA-1 (25uM) for 6 hours and the cells were washed twice with PBS and were allowed to recover in normal culture medium till harvesting, 6- P100V were treated with PRIMA-1 (25uM) for 6 hours and the cells were washed twice with PBS and were allowed to recover in normal culture medium overnight. These cells were subsequently treated with bortezomib (25nM) for 48 hours. O/N: overnight. **F.** Cell viability of JJN3 after 48 hours treatment with either fixed low dosage of PRIMA-1 (10uM), increasing dosage of bortezomib or combination of PRIMA-1 (10uM) with the corresponding bortezomib dosage on the x-axis. **G.** JJN3 were treated with either PRIMA-1 (25uM), or bortezomib (5nM) or combination of both. Cells were harvested to check for the protein expression of the UPR markers. **H.** The mRNA level of UPR markers in JJN3 after treatment with either PRIMA-1 (25uM), or bortezomib (5nM) or combination of both. * p < 0.05, ** p < 0.01.

When we treated primary patient samples with PRIMA-1, they experienced a gradual growth inhibition with increasing dosage (Figure 7A). Consistent with HMCL observations, absence of p53 expression apparently conferred higher amount of apoptosis (c-PARP) in both N073 and T019 as compared with N099 and T011 (Figure 7B). This further confirms that even in clinical samples, PRIMA-1's activity is independent of p53 and was more effective in MM cells without p53 expression. Importantly, the cytotoxicity profile in the patient samples also corresponded well with robust UPR activation (Figure 7C).

Bortezomib, being the novel anti-myeloma agent, has also been reported to induce the UPR activation [27, 32]. More importantly, previous studies have suggested that the sensitivity to bortezomib is dependent on a cellular state where UPR is activated [33]. These raised the possibility that if PRIMA-1 can elevate ER stress to induce UPR in myeloma cells, then it could sensitize them to bortezomib and that the combination would be more effective (same concept as Figure 4).

To test this hypothesis, we utilized the previously generated bortezomib-resistant HMCL, RPMI-8226-P100V. This HMCL was resistant to bortezomib of up to 50nM (Supplementary Figure S7A), whereas its parental cell line, RPMI-8226 has a low IC50 of only 4nM (data not shown). Similar to RPMI-8226, PRIMA-1 treatment was effective in inducing growth inhibition of P100V in a dose-dependent manner (Supplementary Figure S7B). Essentially, when P100V was treated with a combination of PRIMA-1 and bortezomib, we observed an enhanced depression in cell survival (Figure 7D). Combination index (CI) was approximately 1.0, suggesting an additive effect for both drugs. This set of data highlighted not only the efficacy of PRIMA-1 in targeting bortezomib resistant cases, but also underlined the likelihood of PRIMA-1 restoring bortezomib sensitivity in myeloma.

To probe into this possibility, P100V was pre-treated with PRIMA-1 and was then allowed to recover for 6 hours before they were being incubated with bortezomib (Figure 7E). This strategy was intended to elevate the basal ER stress and disrupt the UPR equilibrium first before exposing the cells to solely bortezomib. Indeed, pre-treatment with PRIMA-1 caused an increased rate of apoptosis in the bortezomib only-treated cells (compare Figure 7E-5 and 7E-6), indicating that the effect seen in 7E-6 was solely due to the actions of bortezomib since PRIMA-1 has been completely rid of (from the overnight recovery). This underlines the potential re-sensitizing role of PRIMA-1 to bortezomib.

To further establish the importance of PRIMA-1 and bortezomib partnership, drug combination study was then extended to other HMCLs. In JJN3, a low dosage treatment of single agent PRIMA-1 (10uM) and bortezomib rendered the cells to have 20% and 30% of reduction in cell viability, respectively. Strikingly, combination treatment of both drugs led to significantly reduced cell survival (Figure 7F). Additive effects were recorded at the concentrations tested, with CI~1. To determine if the enhanced cytotoxicity was the result of the amplified UPR, we assessed the UPR markers upon combination treatment. True enough, concomitant treatment significantly intensified the expression of HSP70, CHOP, GADD34 and NOXA relatively to the single-agent-treated-cells (Figure 7G and 7H).

Similarly, combination treatment was also performed in the NCI-H929 (p53-WT) and RPMI-8226 (p53-Mut) and the sensitivity of the cells to PRIMA-1 was efficiently increased in the presence of bortezomib (Supplementary Figure S7C), suggesting that PRIMA-1 could co-operate with the latter for enhanced cytotoxicity, presumably by magnifying the UPR activity.

## DISCUSSION

PRIMA-1 is a first-in-class mutant-p53-reactivating agent that has made its way to the Phase I/II clinical trials [15, 34]. This drug demonstrated proof-of-concept that mutant p53 can be rescued and restored to the function of a WT in killing cancer cells. Despite its well established efficacy in cancer cells with p53 mutation, other studies have suggested p53-independent mechanisms. Cells from osteosarcoma, prostate cancer and hepatocellular carcinoma of either p53-WT or p53-null status, were efficiently killed by PRIMA-1 [9, 35, 36]. Of important relevance, this drug was also reported to be effective in blood malignancies, irrespective of p53 status, in AML, B-CLL and MM [16, 17, 37]. Nonetheless, its mechanism of action has yet to be clearly elucidated. These reports collectively erected an important platform for further interrogation of the p53-independent mechanisms of PRIMA-1. This is crucial given that PRIMA-1 has been described to potentially activate various apoptosis-inducing pathways, giving rise to its essential multi-faceted functions [38].

Our study unveiled the perturbation of the UPR as a novel mechanism by which PRIMA-1 exerts its cytotoxicity in MM. We saw a robust induction of various ER stress markers, namely, HSP70, CHOP, GADD34 and NOXA in HMCLs and patient samples. Additionally, we also observed a global reduction of protein synthesis, consistent with the function of UPR in re-establishing intracellular protein homeostasis, thus, implicating that PRIMA-1 cytotxicity in MM cells was to a large extent working through the UPR pathway. Attesting to our findings, the association of ER stress with PRIMA-1 has actually been briefly reported before in osteosarcoma, nonetheless, it was a mutant-p53-dependent event [38]. Importantly, our finding was relevant in the context of MM because these malignant plasma cells being involved in excessive immunoglobulin production, naturally has an almost-saturated level of cytoprotective UPR mechanism, thus, they are more easily sensitized to the induction of terminal UPR in response to additional ER stress by exogenous agents such as PRIMA-1.

We have demonstrated that the intracellular ER stress level was critical for PRIMA-1's therapeutic mechanism in MM. Only in the presence of tunicamycin to pre-induce unfolded protein accumulation that the less sensitive HMCL (U266) could undergo significantly higher PRIMA-1-induced-apoptosis, accompanied by concomitant increase of UPR markers. This essentially means that tunicamycin sets the base for PRIMA-1 to further impose ER stress, tipping the homeostasis balance in favour of apoptosis. In relation to this, we propose that a certain threshold of ER stress needs to be surpassed for PRIMA-1 to be effective. Supporting this hypothesis, bortezomib (acting similiarly as PRIMA-1), was also apparently more effective in targeting MM cells with higher immunoglobulin production (higher basal ER stress) [32].

Previously, p73 was identified to be important for PRIMA-1 cytoxicity in myeloma [16]. We further extended this finding by demonstrating that p73 induction could result from the down-regulation of DNMT1, leading to a subsequent demethylation of *TP73,* and a sustained UPR activity. The fact that our p53-null study models were most sensitive to the drug makes our data all the more relevant as absence of p53 could trigger its family member, p73, to act as a surrogate TSG. Supporting our findings, a recent study on thyroid cancer has reported that PRIMA-1Met was indeed able to induce global DNA demethylation and upregulation of various tumor suppressors [39].

Another study in MM has revealed the association between PRIMA-1^Met^ and reactive oxygen species (ROS) [17]. Consistently, we also found that ROS is induced upon PRIMA-1 treatment but it was a downstream event of UPR. Evident elevation of ROS marker was observed only at the 16^th^ hour onwards upon PRIMA-1 treatment (Supplementary Figure S5C), whereas UPR markers were already upregulated at the 6^th^ hour (Figure 2C). Given that ROS has been consistently linked to ER stress-induced apoptosis in cancer [40, 41], it is conceivable that all these factors are inter-connected to form a dynamic network in driving the eventual PRIMA-1 cytotoxicity in MM (Supplementary Figure S8).

At the molecular level, PRIMA-1 was reported to restore the functional p53 protein conformation and phenotypic activity through its active compound, methylene quinuclidinone (MQ) that binds to and forms adduct with the cysteine residues (Cys) of mutant p53 proteins [10]. While we did not observe this phenotype in the p53-mutant MM cells, it is plausible that MQ could also bind to other proteins (such as p73 in our case), since all intracellular proteins potentially contain Cys, and cause modifications, which may then affect cellular milieu and protein homeostasis. We speculate that this could at least, in part, explain why PRIMA-1 could trigger UPR, bringing about p53-independent-apoptosis. Supporting this theory, the ability of PRIMA-1/MQ binding to other proteins (TrxR1 and glutathione) has been reported previously [42, 43].

Regarding why PRIMA-1 was more efficacious in the absence of p53 expression, it is likely that p53, being the guardian of the genome, will protect genome integrity by suppressing stress, including that in the ER, that is required by PRIMA-1 to kill the MM cells. Furthermore, as p73 was found to be essential in its mechanism of action, the presence of p53 may possibly suppress the effects of p73. This hypothesis is in line with mutant p53′s gain-of-function role of binding to TA-p73, rendering the latter functionally defective [44, 45]. Nevertheless, these possibilities have to be carefully interrogated and is beyond the scope of current study.

Clinically speaking, since we have shown that myeloma cells lacking p53 expression were more sensitive to the drug, we strongly feel that the high risk 17p13(del) patients who experience p53 haploinsufficiency and total abolishment of p53 expression [7] could potentially benefit from this drug. Our finding corroborates with a previous observation in AML in which hemizygous 17p13(del) subgroup was more sensitive to PRIMA-1 [46]. Moreover, the fact that PRIMA-1 was effective against all the HMCLs tested, irrespective of their p53 status (albeit at higher concentrations), really underscores its versatility in targeting myeloma cases with different genetic alterations.

It is understandable if one is concerned about the relatively high PRIMA-1 dosage required for its efficacy. However, previous reports have ruled out its toxicity effects in the xenograft system (the mice were administered with high doses of the drug, up to 100mg/kg) [11, 12] as well as in the bone marrow and hematopoetic progenitor cells [15, 16]. Of paramount importance, the first-in-human study with PRIMA-1Met has reported 60mg/kg as the maximum tolerated dose, which corresponds to a maximum plasma concentration of about 300uM [15]. In our study, we already saw a complete eradication of the MM cells at 100uM in all the HMCLs tested. This simply means that efficient killing of MM can already be achieved at a dosage that is substantially lower than the reported maximum tolerated dose.

In addition, we have shown that PRIMA-1's combination with bortezomib could induce significant growth inhibition in HMCLs. Since bortezomib has also been associated with the involvement of UPR in MM [27, 47], we believe that combination of both drugs could cause major perturbation to the UPR, thus, driving the cells into major pro-apoptotic state. Importantly, we observed that bortezomib-resistant cells were also sensitive to PRIMA-1. As the clinical efficacy of bortezomib is often impeded by eventual resistance, our finding denotes that PRIMA-1 may possibly be an important bortezomib re-sensitizing agent, however, the optimum dosage of both drugs needs to be prudently scrutinised. Although PRIMA-1 has been reported to synergize with various cytotoxic drugs such as dexamethasone, doxorubicin and cisplatin [14, 16, 48], to the best of our knowledge, our study is the first to report the novel efficacy of PRIMA-1-bortezomib combination.

In conclusion, we have unveiled a novel mechanism by which PRIMA-1 could exert its cytotoxicity in MM cells, which is via the UPR/ER stress pathway. This denotes that PRIMA-1's mechanism of action is actually much more diverse than previously thought, underlining its promising role in targeting various tumor suppressor pathways and its versatility in keeping drug resistance at bay. Essentially, the double-edged sword properties of UPR provides a good channel for therapeutic manipulation in cancer. Because high ER stress level remains one of the hallmarks of MM, exploiting this Achilles heel of the disease would represent an attractive therapeutic strategy.

## MATERIALS AND METHODS

### Human myeloma cell lines (HMCLs) and primary myeloma cells

All HMCLs used have been previously characterized [7]. For primary myeloma cells, the blood or bone marrow samples were collected from patients after obtaining informed consent, in accordance with the Declaration of Helsinki. Plasma cells were isolated through Ficoll-Hypaque centrifugation system and were purified with CD138 immunomagnetic beads (Stemcell Technologies, Vancouver, British Columbia, Canada). Culturing conditions are stated in Supplementary Information.

### Drug treatment

All drugs were dissolved in dimethyl sulfoxide (DMSO) and are stored at −20C. PRIMA-1 was purchased from Cayman Chemical (Ann Arbor, MI, USA). Tunicamycin and 5-Azacytidine were bought from Sigma-Aldrich (St Louis, MO, USA). Bortezomib was obtained from the clinic. Cells were seeded at 0.25×10^6^ cells/mL prior to treatment.

### Cell viability and apoptosis assay

Cell viability of MM cells was determined by using MTS (3-(4,5 dimethylthiazol-2-yl)-5-(3-carboxymethoxyphenyl-2-(4-sulfophenyl)-2H-tetrazolium) assay [7]. Rate of apoptosis was checked by using Annexin-V-FITC assay (BD Pharmingen, San Jose, CA, USA) according to manufacturer's protocol. Percentage specific apoptosis was calculated as previously described [7]. Protein levels of cleaved-PARP and cleaved-caspases-8, -9 and -3 were used as a semi-quantitative gauge for apoptosis.

### Gene silencing and overexpression

p53 stable knockdown NCI-H929 has been generated previously [7]. Introduction of either sip73 (Dharmacon, Lafayatte, CO, USA) or pCDNA-p73 (Addgene, Cambridge, MA, USA) was performed using Neon Transfection System (Life Technologies, Carlsbad, CA, USA). Transfection was done at 0.5×10^6^ cells/mL density, followed by drug treatment 48 hours post-transfection.

### Gene expression microarray analysis

Gene expression profiling (GEP) was done on Affymetrix platform (Santa Clara, CA, USA) according to the manufacturer's protocol. Differentially expressed genes of at least 2-folds (p<0.05) upon PRIMA-1 treatment were identified. Only overlapping up/downregulated genes in both HMCLs (JJN3 and KMS11) were considered specific for PRIMA-1 activity.

### Protein and mRNA expression

Protein expression was analysed by standard Western Blot analysis. List of antibodies used is in the Supplementary information. mRNA expression was assessed with Real-Time PCR via Sybr Green (Bio-rad, Hercules, CA, USA) method. Primer sequences used are listed in the Supplementary information.

### XBP1s assay

To evaluate expression levels of XBP1u and XBP1s, RT-PCR analysis was performed with human XBP1 primer sequences [49]. PCR products were visualized on a 2.5% agarose gel.

### Luciferase reporter assay

Cells were transfected with Cignal ERSE (ER stress response element) reporter (Qiagen, Venlo, Limburg, Netherlands) and was treated 24 hours post-transfection. Detailed procedure is described in the Supplementary Information.

### Surface Sensing Of Translation (*SUnSET*) assay

Upon the completion of timepoint treatment, the cells were incubated 30 min in medium containing 1 μM puromycin. Cells were harvested and total protein extraction was performed as per standard protocol. Cell lysates were subjected to conventional Western blotting using anti-puromycin antibody.

### Statistical and bioinformatics analysis

All statistical analyses were done with Independent T-test, assuming normal distribution of mean. IC50s were analyzed using CompuSyn (Combosyn, Inc., Paramus, NJ, USA). Gene Ontology (GO) pathway analysis was conducted using DAVID, v6.7 (Database for Annotation, Visualization and Integrated Discovery) (National Cancer Institute at Frederick, Frederick, MD). All statistical data are presented as mean ± standard deviation.

## SUPPLEMENTARY MATERIALS FIGURES


