# Supplementary material for: PRIMA-1 targets the vulnerability of multiple myeloma of deregulated protein homeostasis through the perturbation of ER stress via p73 demethylation
Source: Oncotarget. 2016 Aug 12;7(38):61806–19. doi: 10.18632/oncotarget.11241 (PMC5308692; doi:10.18632/oncotarget.11241)
Supplement: Supplementary file 1 [file oncotarget-07-61806-s001.pdf]

PRIMA-1 targets the vulnerability of multiple myeloma of deregulated protein homeostasis through the perturbation of ER stress via p73 demethylation

SUPPLEMENTARY INFORMATION

MATERIALS AND METHODS

Cell Culture

All HMCLs were grown at 37°C in a CO_2 incubator.

HMCL	Culturing conditions
NCI-H929	RPMI1640, 10% FBS, 1% Penicillin/streptomycin, 2mM L-glutamine, 0.05mM beta-mecarptoethanol
XG6	RPMI1640, 10% FBS, 1% Penicillin/streptomycin, 2mM L-glutamine, 0.05mM beta-mecarptoethanol, 2nM IL-6
KMS18, JJN3, KMS11, RPMI-8226, U266	RPMI1640, 10% FBS, 1% Penicillin/streptomycin, 2mM L-glutamine
Primary myeloma samples	IMDM medium, supplemented with 20% FBS, 1x L-glutamine, 1x Pennicillin/ Streptomycin, 25mM HEPES, 55uM B-Mercaptoethanol, 10ng/mL interleukin-6, 100ng/mL IGF-1 and 50ng/mL BAFF.

List of antibodies used in Western Blot analysis

Antibodies	Company	Dilution
p53 (DO-1)	Santa-Cruz	1:2000
p21	Cell Signalling	1:1000
MDM2	Santa Cruz	1:2000
PUMA	Cell Signalling	1:1000
NOXA	CalBiochem	1:1000
c-PARP	Cell Signalling	1:2000
Caspase-3	Cell Signalling	1:2000
Caspase-8	Cell Signalling	1:2000
Caspase-9	Cell Signalling	1:2000
Gapdh	Santa Cruz	1:5000
CHOP	Cell Signalling	1:1000
eIF2a	Cell Signalling	1:1000
p-eIF2a	Cell Signalling	1:500
GRP78	Cell Signalling	1:1000
p73	Abcam	1:1000
Gadd34	Abcam	1:1000
P-PERK	Santa Cruz	1:500
BCL-2	Santa Cruz	1:500
MCL-1	Santa-Cruz	1:500
Goat anti-mouse	Santa Cruz	1:5000
Goat anti-rabbit	Santa Cruz	1:5000
Mouse Anti-goat	Santa Cruz	1:5000

List of primary sequence		
**Gene**		**Primer sequences**
NOXA	Forward	5′-GCTGGAAGTCGAGTGTGCTA-3′
	Reverse	5′-CCTGAGCAGAAGAGTTTGGA-3′
GADD34	Forward	5'-AGCGCCCAGAAACCCCTACTCAT-3'
	Reverse	5'-AGACAGCCAGGAAATGGACAGTGA-3'
HSP70	Forward	5′-TGTCGTCCAGCACCCAGGCCAGC-3'
	Reverse	5′-GCTCTTGTTCAGGTCGCGCCCG-3'
CHOP	Forward	5'-AGAACCAGGAAACGGAAACAGA-3'
	Reverse	5'-TCTCCTTCATGCGCTGCTTT-3'
ATF4	Forward	5'-TCTCCAGCGACAAGGCTAA-3'
	Reverse	5'-CAATCTGTCCCGGAGAAGG-3'
GRP78	Forward	5'-CCAAGAGAGGGTTCTTGAATCTCG-3'
	Reverse	5'-ATGGGCCAGCCTGGATATACAACA-3'
p73	Forward	5'-CTCCCCGCTCTTGAAGAAAC-3'
	Reverse	5'-GTTGAAGTCCCTCCCGAGC-3'
GAPDH	Forward	5'-GGCTGTGGGCAAGGTCATCCCTGAG-3'
	Reverse	5'-GTCGCTGTTGAAGTCAGAGGAGACCACCTG-3'

Luciferase reporter assay

JJN3 and KMS11 were transfected with Cignal ERSE (ER stress response element) reporter (Qiagen), a luciferase reporter plasmid which has an ER stress response element (CCAAT(N9)CCACG) upstream of the firefly luciferase gene. 24 hours after the transfection, the cells were washed with PBS and were then treated with DMSO, PRIMA-1 and/or tunicamycin (positive control) for another 16 hours, after which the cells were harvested and were analyzed with the Dual Luciferase system (Promega). Renilla luciferase construct acts as an internal control. Data was presented as ratios, with the activity of cells transfected with control vector arbitrarily set at 1.

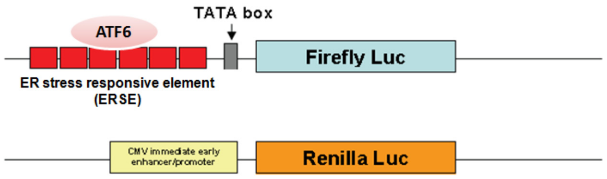

Firefly luciferase system for the detection of ATF6 binding to ER stress responsive elements on its direct targets.

Reactive Oxidative Stress (ROS) detection assay

1 million cells were DMSO- or PRIMA-1-treated for 6 hours. The cells were then harvested and washed with PBS twice. After that, 200 uL of CM-H_2DCFDA (an indicator for ROS) conjugated with FITC dye (Life Tech, Carlsbad, CA, USA) was added into the cells to a final concentration of 2.5 uM, and the solution was incubated at 37°C for 30 minutes. Following that, the dye was washed away twice with PBS. After the final wash, the cells were re-suspended in PBS and were transferred to the FACS tube through filter mesh. ROS-positive cells were quantified with BD FACs DIVA software.

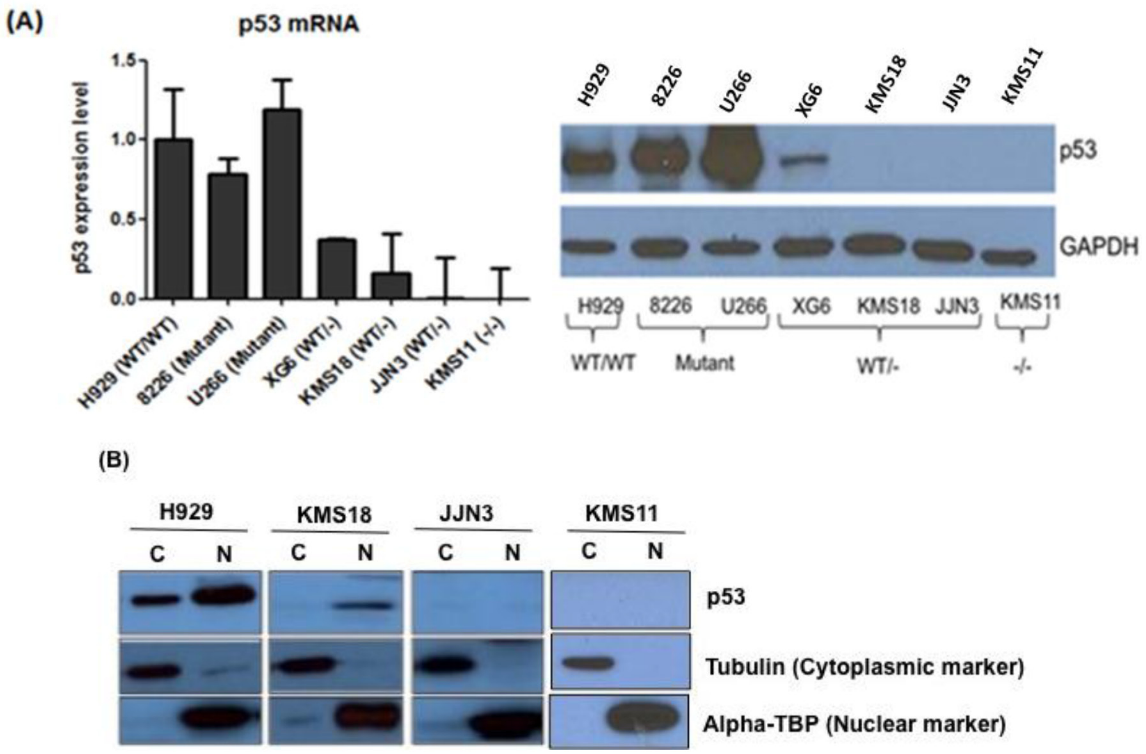

Supplementary Figure S1: A. Basal p53 mRNA and protein level in human myeloma cell lines. The p53 genotype of each cell lines are stated. WT/WT: double copies of Wild type *TP53*, WT/-: monoallelic deletion, Mutant: *TP53* mutation at the DNA binding domain, -/-: *TP53* null. Most sensitive cell lines, JJN3 and KMS11 were lacking p53 expression at both the mRNA and protein level. **B.** Western blot analysis on nuclear and cytoplasmic extracts of H929, KMS18, JJN3 and KMS11. Fig. S1A showed that KMS18 had no p53 protein expression but it was not as sensitive to PRIMA-1 as JJN3 and KMS11. Fig. S1B showed evidence of p53 expression at the nuclear level in KMS18 but not in the sensitive cell lines, JJN3 and KMS11, indicating that the presence of p53 does indeed mask the sensitivity of MM cells to PRIMA-1. C: Cytoplasmic fraction, N: Nuclear fraction.

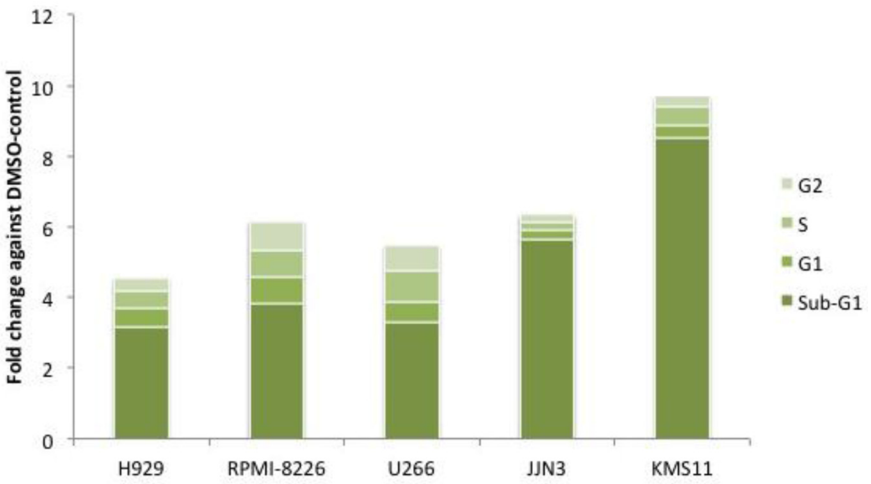

Supplementary Figure S2: Cell cycle analysis in response to PRIMA-1 treatment. Fold change of each phases in the cell cycle in PRIMA-1-treated sample against DMSO control. JJN3 and KMS11 recorded highest increase in sub-G1 population.

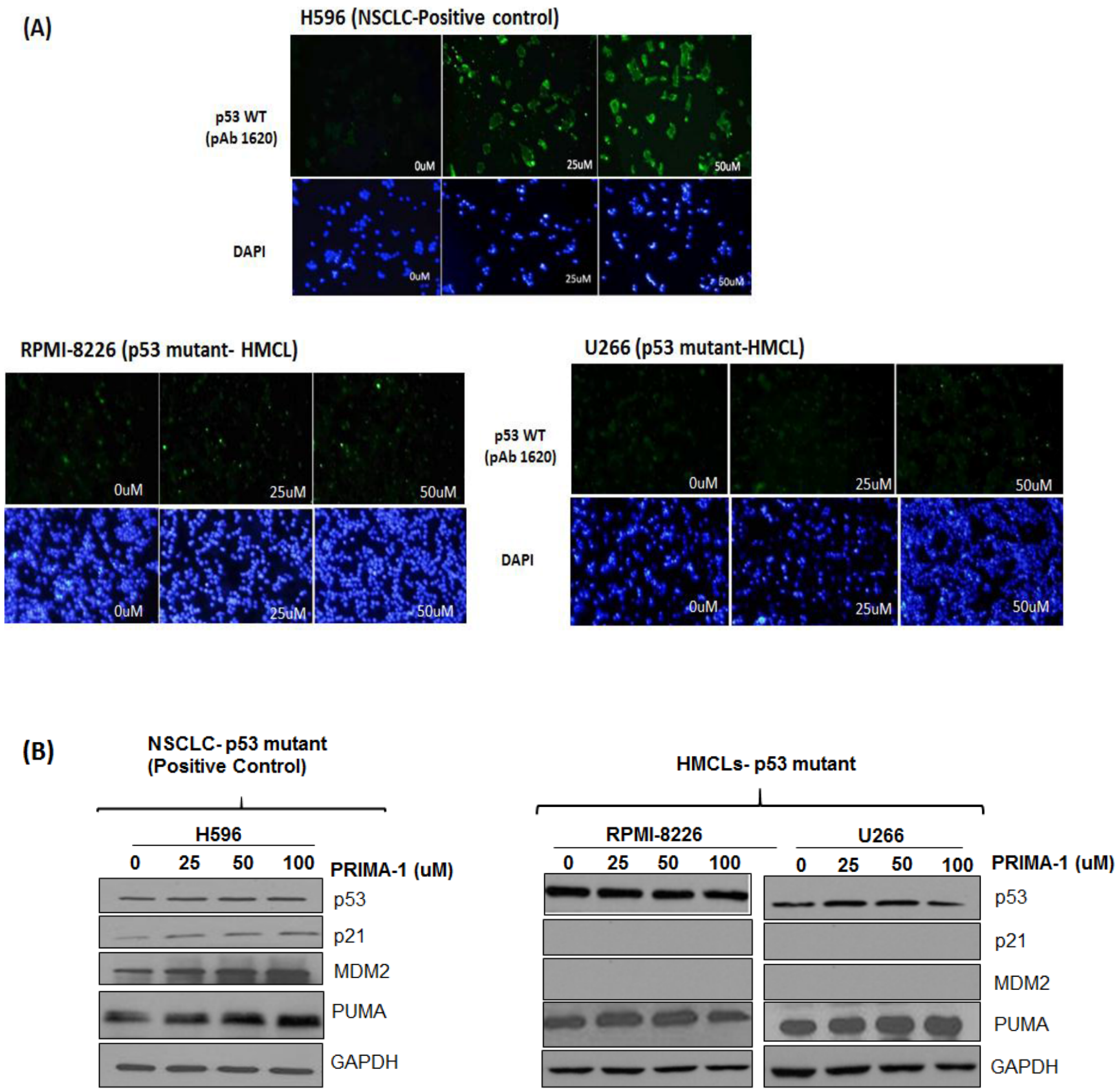

Supplementary Figure S3: A. Immunofluoresence staining for WT p53 protein before and after PRIMA-1 treatment (24 hours) in a NSCLC cell line (H596) and two HMCLs (RPMI-8226 and U266). No reconstitution of WT p53 conformation in HMCLs as detected by p53 WT-specific antibodies. PRIMA-1 dosage used was 0uM, 25uM and 50uM. Original magnification x100. NSCLC: non-small cell lung carcinoma. **B.** Western blot analysis for p53 pathway activity in p53-mutant cell lines (H596, RPMI-8226 and U266) after treatment for 24 hours. No restoration of p53 transcriptional function in HMCLs. p53 downstream targets p21, MDM2 and PUMA were not induced upon PRIMA-1 treatment.

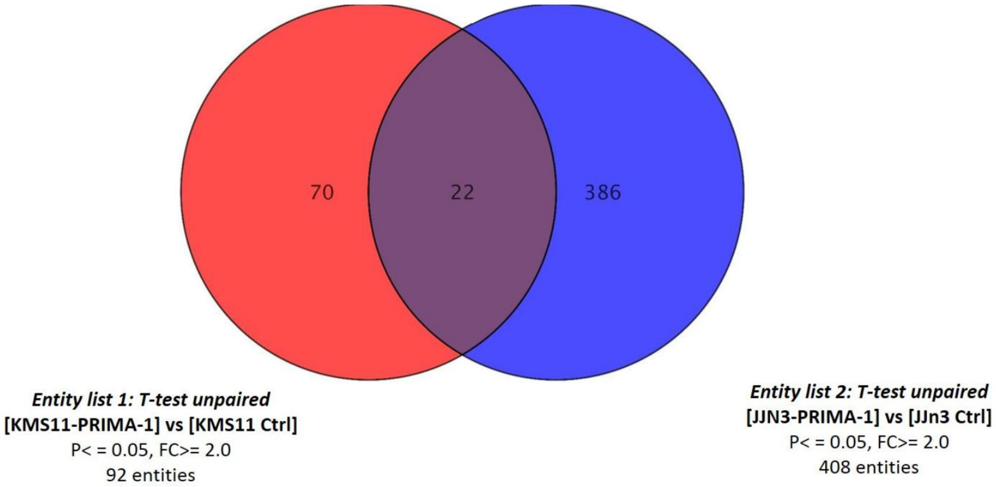

Supplementary Figure S4: The number of differentially expressed genes (either up-regulated or down-regulated) in PRIMA-1-treated KMS11 and JJN3 versus DMSO control as analysed by Gene Expression Microarray Profiling (Affymetrix). Twenty-two overlapping genes were identified between the two cell lines.

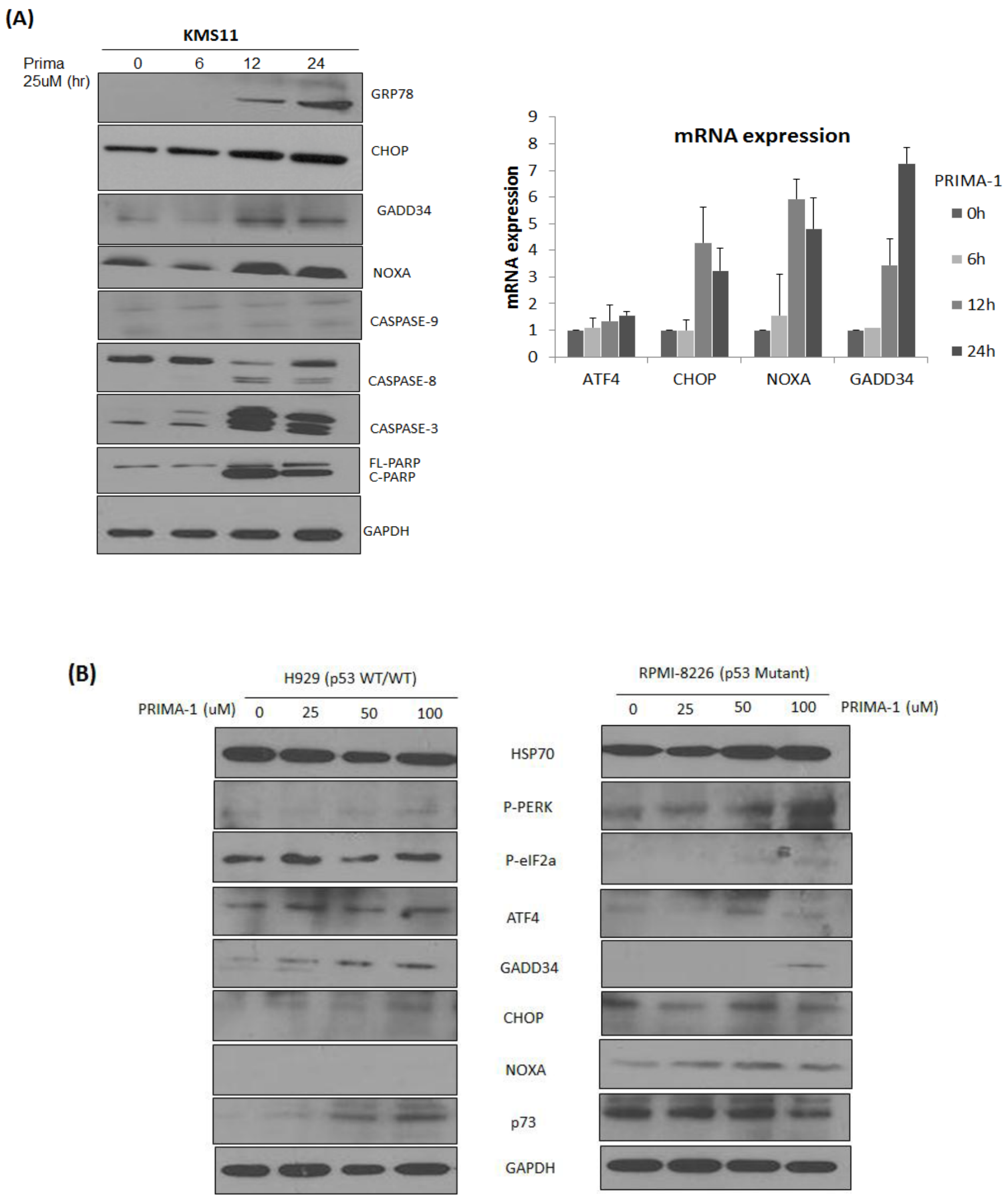

Supplementary Figure S5: A. ER stress and apoptotic markers in KMS11, before and after PRIMA-1 treatment at the protein and mRNA levels. ER stress and apoptotic markers were concurrently upregulated. **B.** Change of protein expression of UPR markers in response to PRIMA-1 treatment in H929 and 8226. Mild up-regulation of some of the markers are observed upon PRIMA-1 exposure. **C.** PRIMA-1 treatment induces activation of ROS pathway as indicated by the increase of CM-H_2DCFDA (ROS marker) in a time-dependent manner. (*Continued*)

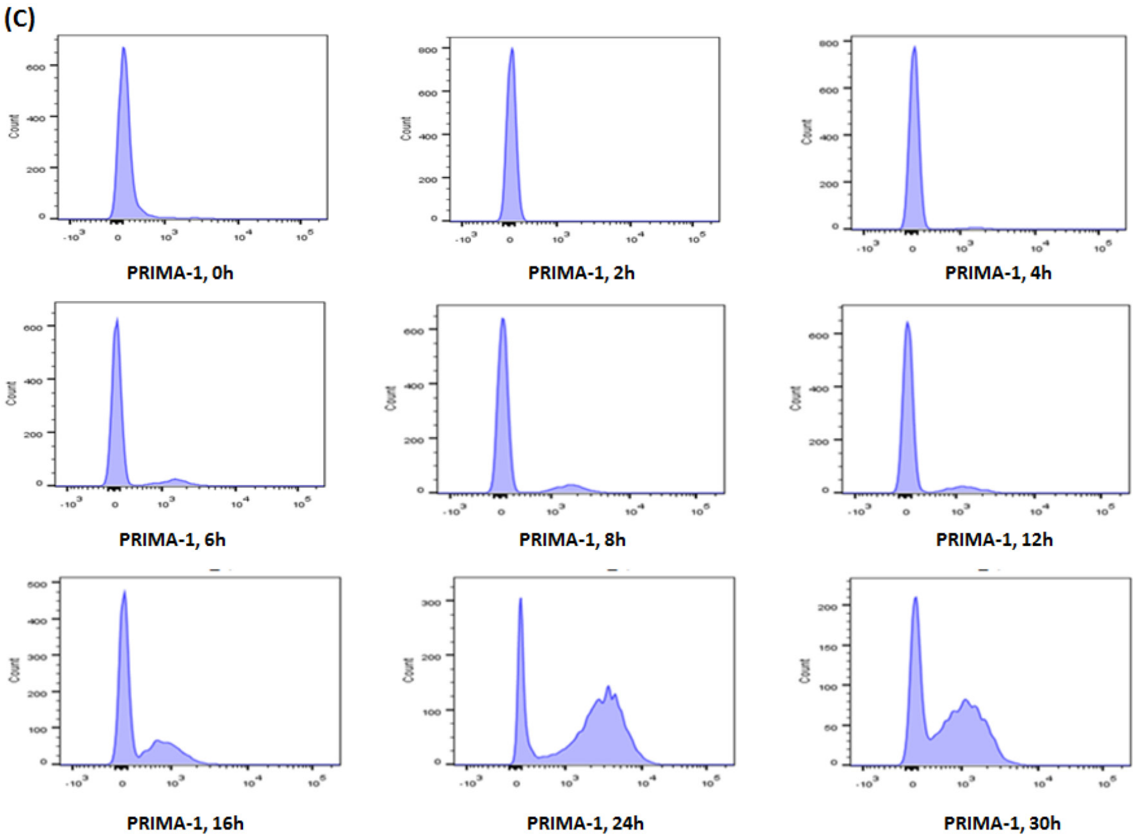

Supplementary Figure S5: C. PRIMA-1 treatment induces activation of ROS pathway as indicated by the increase of CM-H_2DCFDA (ROS marker) in a time-dependent manner.

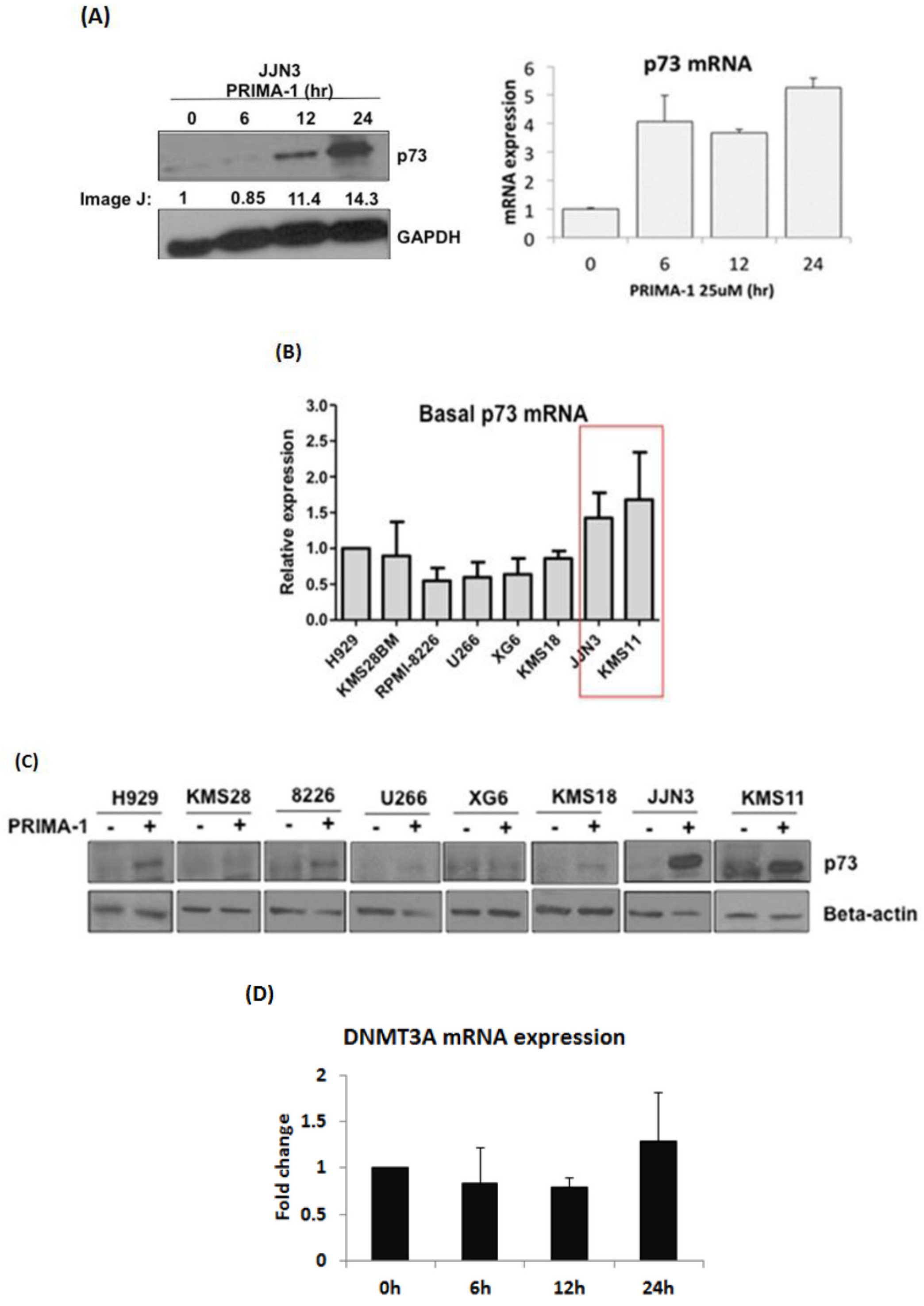

Supplementary Figure S6: A. p73 was upregulated at both protein and mRNA level when JJN3 was treated with PRIMA-1 (25uM), in a time dependent manner. **B.** Basal *TP73* mRNA expression in HMCLs. Cells with higher sensitivity to PRIMA-1 (JJN3 and KMS11) have relatively higher levels of p73 mRNA as compared to cells with lower sensitivity. **C.** p73 protein expression before and after PRIMA-1 treatment (25uM). Significant upregulation of p73 was seen in JJN3 and KMS11. **D.** mRNA expression of DNMT3A after treatment of JJN3 (25uM) with PRIMA-1 at different time points.

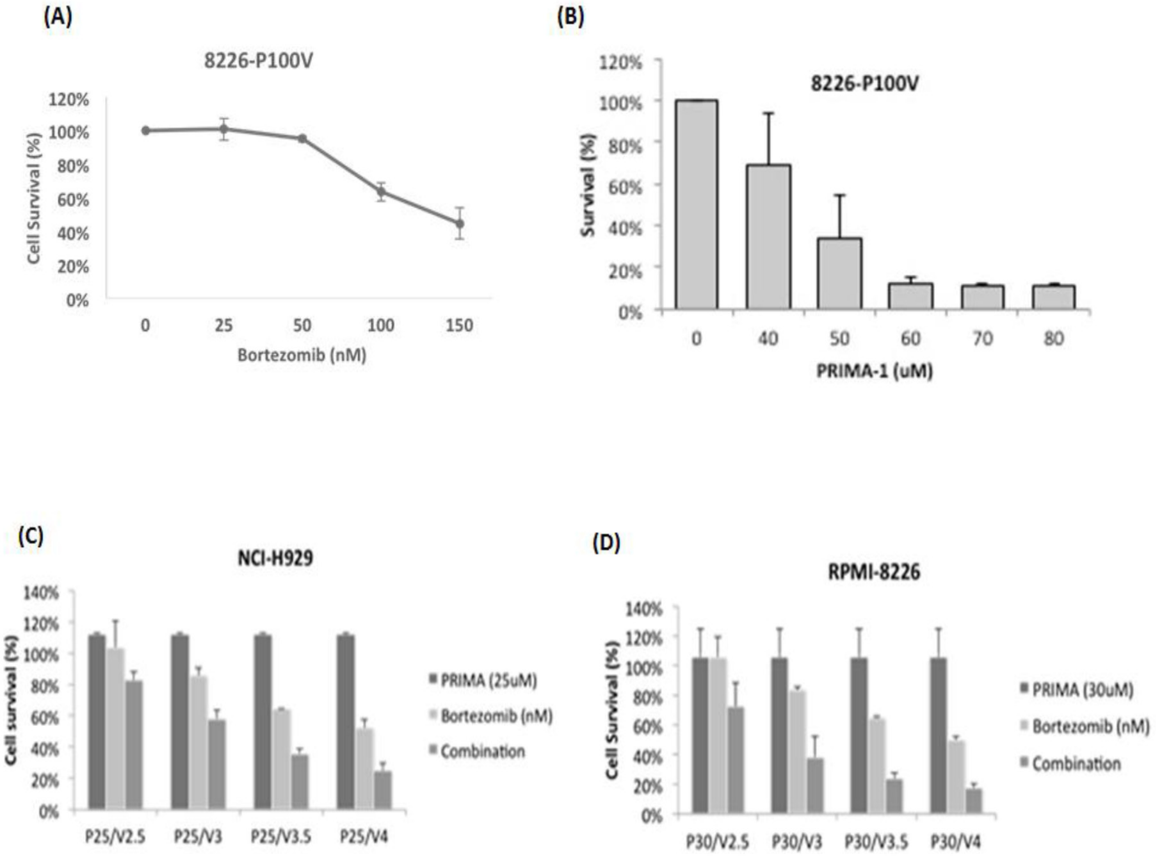

Supplementary Figure S7: A. MTS assay of 8226-P100V treated with bortezomib of increasing dosage. This cell line was generated to be resistant to bortezomib. Its IC50 for bortezomib was ~100nM. **B.** MTS assay of 8226-P100V treated with PRIMA-1 of increasing dosage. PRIMA-1 treatment causes dose-dependent decrease of cell viability in 8226-P100V. **C** and **D.** Combination treatment of PRIMA-1 and bortezomib (48 hours) in NCI-H929 (C) and RPMI-8226 (D). Significant reduction in cell survival upon combination treatment in both HMCLs. P at the x-axis represents the dosage of PRIMA-1 in uM and V represents bortezomib dosage in nM.

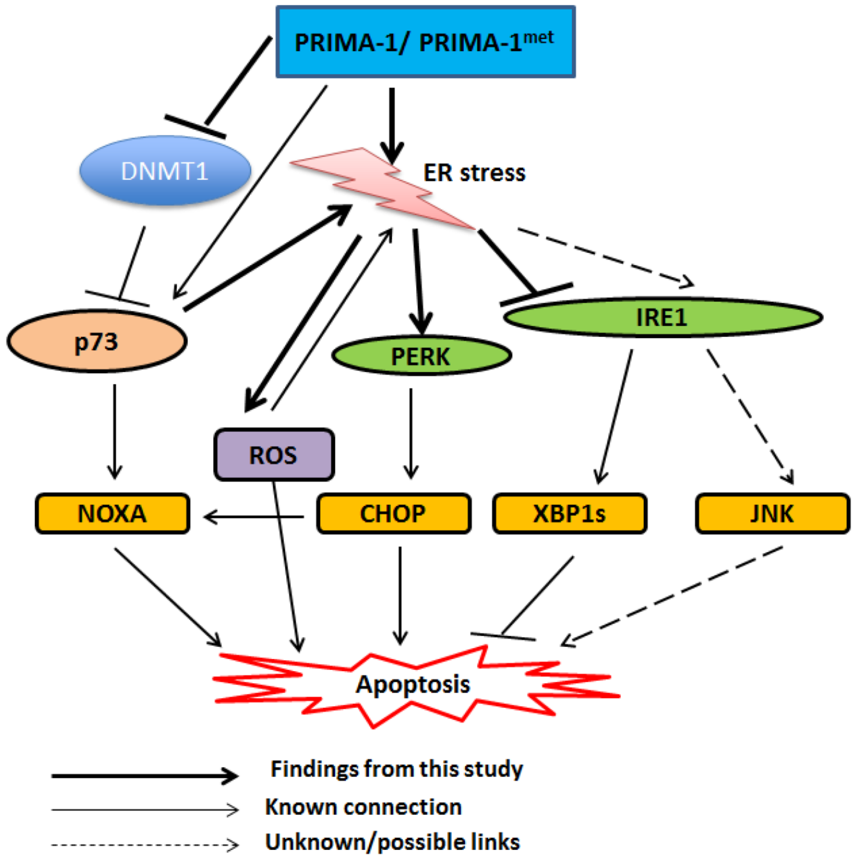

Supplementary Figure S8: The potential connections between p73-ER stress-ROS upon PRIMA-1 induction in MM.

Supplementary Table S1: IC50 of PRIMA-1 in all the HMCLs

HMCL	P53 STATUS	IC50 (PRIMA-1)(uM)
H929	WT/WT	54.8458
KMS28BM	Mutant	88.8962
RPMI-8226	Mutant	71.6141
U266	Mutant	70.3264
XG6	WT/-	83.1861
KMS18	WT/-	65.3732
**JJN3**	**WT/- (no p53 expression)**	**32.2331**
**KMS11**	**-/- (no p53 expression)**	**16.3025**

IC50 of PRIMA-1 in all the HMCLs tested. It is noted that (bolded) JJN3 and KMS11, both with no p53 expression demonstrated the lowest IC50. HMCL: Human Myeloma Cell Line; WT: Wild Type; -: Allelic Loss

Supplementary Table S2: IC50 of PRIMA-1Met in all the HMCLs

HMCLs	IC50 (PRIMA-1Met) (uM)
RPMI-8226	24.5725
KMS28	23.5472
U266	21.9627
KMS18	11.5499
H929	9.04681
**JJN3**	**4.8216**
**KMS11**	**2.04472**

IC50 of PRIMA-1Met in the HMCLs. It is noted that (bolded) JJN3 and KMS11, both with no p53 expression demonstrated the lowest IC50.

Supplementary Table S3: Gene Ontology Pathway Analysis for JJN3 and KMS11

Pathway	*P value*	
	**JJN3**	**KMS11**
Response to unfolded protein	1.90E-06	6.12E-08
Response to topologically incorrect protein (misfolded protein)	2.99E-06	8.21E-08

Gene ontology pathway analysis of GEP data by using DAVID revealed two significantly enriched and highly relevant pathways in MM.
